# Exploring the intersections of college student poverty, grief, and racial/ethnic identity: a scoping review protocol

**DOI:** 10.1186/s13643-023-02407-x

**Published:** 2023-12-14

**Authors:** Sarah Elizabeth Garza-Levitt, Mary M. McFarland, Kimberly Ponce Gonzalez, Katherine P. Supiano

**Affiliations:** 1 The University of Utah College of Social Work, 395 South 1500 East, Salt Lake City, UT 84112 USA; 2 The University of Utah Eccles Health Sciences Library, 10 North 1900 East, Salt Lake City, UT 84112 USA; 3 The University of Utah College of Nursing, 10 South 2000 East, Salt Lake City, UT 84112 USA

**Keywords:** College students, Bereavement, Death, Grief, Grieving, Ethnicity, Race, Poverty, Students of color, Socioeconomic status

## Abstract

**Background:**

College students who experience the negative impact of poverty, such as food, financial, and housing insecurity, are at higher risk for poor academic performance. One recent study examined grief in a college student sample and found students with a diverse racial or ethnic background were more likely to experience prolonged grief disorder, however, did not examine poverty in their sample. To date, no known reviews have examined poverty by racial and ethnic identity and the experience of grief due to the death of a family member or friend, and no reviews have examined how these three factors relate to interventions designed to support student academic performance and degree completion.

**Methods:**

Our aim is to map the primary literature reporting on college students of any age who identify or are identified as non-white racial or ethnic groups who experience poverty and grief due to the death of a family member or friend. The mapping strategy includes extracting the various types of interventional support of academic performance and degree completion delivered from campus or community services in any geographic setting worldwide. We will conduct our scoping review with guidance from the latest version of the *JBI Manual for Evidence Synthesis*. Utilizing the framework as outlined by Arksey and O’Malley, we will conduct our scoping review with Arksey’s five stages: (1) identifying the research question, (2) identifying relevant studies, (3) study selection, (4) charting the data, and (5) collating, summarizing, and reporting the results. For transparency and reproducibility, we will adhere to the PRISMA reporting guidelines.

**Discussion:**

The purpose of this scoping review is to map the primary literature reporting college students, regardless of their age, who belong to non-white racial or ethnic groups and face poverty and grief resulting from the loss of a family member or friend. This analysis includes mapping the various types of intervention and support available both on and off campus, in any global setting, with the aim of enhancing academic performance and facilitating degree completion. The results of this review may inform the further research needed in this area to help prevent poor academic performance and dropout for many vulnerable college students. The results may be of value, particularly to college administrators developing prevention and interventional programs to support college student success.

**Systematic review registration:**

Open Science Framework (https://osf.io/enuwt).

**Supplementary Information:**

The online version contains supplementary material available at 10.1186/s13643-023-02407-x.

## Background

College students who experience the negative impact of poverty, such as food, financial, and housing insecurity, are at higher risk for poor academic performance [1–9]. Additionally, students with an underrepresented racial or ethnic minority identity have a higher likelihood of experiencing poverty [10, 11]. These college students have also been disproportionally impacted by COVID-19, with pandemic-related deaths exceeding 6.97 million worldwide [12, 13]. Consequently, students with an underrepresented racial or ethnic minority identity are more likely to experience poverty and grief simultaneously. One recent study examining college student grief found that students with an underrepresented racial or ethnic minority identity were more likely to experience prolonged grief disorder (PGD). They found that “the rate of PGD among Black students (16.7%) was twice that of white students (8.8%). The rates of PGD among Asians (15.8%) and Hispanics (10.5%) were also higher than that of whites” [14]. However, poverty was not examined in their sample. To date, no known reviews have examined poverty among college students with an underrepresented racial and/or ethnic identity who experience grief due to the death of a family member or friend, and no reviews have examined how these three factors relate to interventions designed to support student academic performance and degree completion.

## Methods/design

Our aim is to map the primary literature reporting on college students of any age who identify or are identified as non-white racial or ethnic groups who experience poverty and grief due to the death of a family member or friend. The mapping strategy includes extracting the various types of interventional support of academic performance and degree completion delivered from campus or community services in any geographic setting worldwide.

Preliminary searches of MEDLINE (Ovid) 1946–2022, the Cochrane Database of Systematic Reviews (Wiley), and *JBI Evidence Synthesis* were conducted (SEGL) on September 26, 2022. On November 07, 2022, searches for existing reviews or protocols were conducted (MMM) in the following databases and journals: PubMed (pubmed.gov), Epistemonikos (epistemonikos.org), Cochrane Library (Wiley), PROSPERO (www.crd.york.ac.uk/PROSPERO), Open Science Framework (osf.io), *JBI Evidence Synthesis*, *International Journal of Evidence-Based Healthcare*, and *Campbell Systematic Reviews*. No current or underway systematic or scoping reviews on the topic were identified. The scoping review protocol has been registered within the Open Science Framework (pre-registration number: [https://osf.io/enuwt]).

We will conduct our scoping review with guidance from the latest version of the *JBI Manual for Evidence Synthesis* [15]. Utilizing the framework as outlined by Arksey and O’Malley, we will conduct our scoping review with Arksey’s five stages: (1) identifying the research question, (2) identifying relevant studies, (3) study selection, (4) charting the data, and (5) collating, summarizing, and reporting the results [16]. For transparency and reproducibility, we will adhere to the PRISMA-S and PRISMA-ScR reporting guidelines, and mapping will be done using PRISMA-P [17–20] (see Additional file 1).

We will use the PCC model from the JBI Manual to organize our research question for our scoping review [21].

- Population: College students of any age with racial or ethnic identities other than white.
- Concept: Experiences of poverty and grief due to the death of a family member or friend, as well as interventional support for academic performance and degree completion.
- Context: Any geographic setting worldwide. Interventional support can be delivered from campus or community services, on- or off-campus locations.

### Research question

What has been reported in the primary literature on the intersections of poverty and grief due to the death of a family member or friend in college students of any age who identify or are identified as non-white racial or ethnic groups, and what interventions are delivered to support academic performance and degree completion?

### Information sources and search strategy

An information specialist (MMM) will develop the search strategies using a combination of keywords and database subject headings for the primary databases from sentinel studies and team feedback and then translate the strategy to the other selected databases. Library colleagues will peer review the strategy according to PRESS guidelines [22]. Databases will include the following: MEDLINE (Ovid) 1946–2023, CINAHL Complete (Ebscohost) 1937–2023, APA PsycINFO (EBSCOhost) 1872–2023, Academic Search Ultimate (EBSCOhost) 1965–2023, ERIC (EBSCOhost) 1966–2023, Education Database (ProQuest) (1988–2023), Dissertations & Theses Global (ProQuest) 1861–2023, Sociological Abstracts (ProQuest) 1952–2023, and Scopus (Elsevier) 1970–2023. The search strategy was developed for our primary database MEDLINE and will be translated to the other selected databases (see Additional file 2). Citation management and duplicate detection and removal will be accomplished with EndNote (Clarivate Analytics). We will check references of our included studies for additional reports meeting our inclusion criteria. Grey literature is as follows: Including research and committee reports, government reports, university reports, and conference papers for the last 5 years from the Government Accountability Office, www.gao.gov, and The Hope Center for College, Community and Justice, Temple University, hope.temple.edu.

#### Eligibility criteria

We will use Covidence (Veritas Health Innovation), an online systematic reviewing platform, to screen and select studies.

#### Inclusion criteria

- Population: College students of any age with racial or ethnic identities other than white.
- Concept: Experiences of poverty and grief due to the death of a family member or friend, as well as interventional support for academic performance and degree completion.
- Context: Any geographic setting worldwide. Interventional support can be delivered from campus or community services, on- or off-campus locations.
- Types of studies: All experimental or observational studies with qualitative or quantitative data.

#### Exclusion criteria

Types of studies: Nonacademic media sources (e.g., blogs, news articles, etc.).

### Screening and selection procedure

Two reviewers (SEGL, KPG) will independently screen titles and abstracts and then independently review full text for inclusion. When no consensus can be reached between the two reviewers, a third reviewer (KPS) will be the deciding vote.

### Data extraction

Two reviewers (SEGL, KPG) will pilot our data charting form using sentinel articles prior to the finalization of the protocol. Two reviewers (SEGL, KPG) will then independently extract and chart the data from included studies using Covidence (Veritas Health Innovation). Data extracted from the included studies align with the population (college students of any age with racial or ethnic identities other than white); concept (experiences of poverty and grief due to the death of a family member or friend, as well as interventional support for academic performance and degree completion); and context (any geographic setting worldwide. Interventional support can be delivered from campus or community services, on- or off-campus locations) (see Additional file 3). The data charting form will be revised as required during the data extraction process. Revisions will be reported in the scoping review manuscript. Any conflicts between the two reviewers will be resolved through discussion, or a third reviewer (KPS) will be the deciding vote.

### Quality assessment

In compliance with scoping review methodology, no quality assessment of included studies will be conducted, as our goal is to rapidly map the literature.

### Analysis of evidence

Results from this scoping review will be presented as a descriptive summary (narrative). Additionally, results for the population, concept, and context will be presented in a table, figure, and/or graphical form.

### Dissemination of results

The results will be disseminated through a peer-reviewed publication, conference presentations, and/or a one-day stakeholder meeting. Any changes from the scoping protocol methodology will be acknowledged and defined in the manuscript.

## Discussion

The purpose of this review is to map the primary literature reporting college students, regardless of their age, who belong to non-white racial or ethnic groups and face poverty and grief resulting from the loss of a family member or friend. This analysis includes mapping the various types of intervention and support available both on and off campus, in any global setting, with the aim of enhancing academic performance and facilitating degree completion. The results of this review may inform the further research needed in this area to help prevent poor academic performance and dropout for many vulnerable college students. The results may be of value, particularly to college administrators developing prevention and interventional programs to support college student success. While we understand that this review will only include publications in English, the research question is broad enough to encompass a substantial amount of existing literature. It is important to note that the purpose of a scoping review is not to assess the quality of evidence in a formal manner. Therefore, this review will focus on providing descriptive accounts of the included publications and highlighting any differences in their quality. To minimize the potential for selection bias, we will have multiple members of the research team independently review the data. This review will contribute to the literature base on college student poverty and create greater awareness of the needs of racially and ethnically diverse students experiencing poverty and grief due to death.

### Supplementary Information

**Additional file 1.** PRISMA- P 2015 Checklist.

**Additional file 2.** Ovid MEDLINE(R) Search Strategy.

**Additional file 3.** Draft Data Chart.

## Data Availability

All review data generated or analyzed will be included in the published scoping review article and can be made available upon request.

## Abbreviations

JBI: Joanna Briggs Institute
PRISMA-S: Preferred Reporting Items for Systematic reviews and Meta-Analyses literature search extension
PRISMA-ScR: Preferred Reporting Items for Systematic reviews and Meta-Analyses extension for Scoping Reviews Checklist
PRISMA-P: Preferred Reporting Items for Systematic Review and Meta-analysis Protocol
PCC: Population, concept, and context
