# Supplementary material for: Exploring the intersections of college student poverty, grief, and racial/ethnic identity: a scoping review protocol
Source: Syst Rev. 2023 Dec 14;12:235. doi: 10.1186/s13643-023-02407-x (PMC10720161; doi:10.1186/s13643-023-02407-x)
Supplement: Supplementary file 2 — Additional file 2. Ovid MEDLINE(R) Search Strategy. [file 13643_2023_2407_MOESM2_ESM.pdf]

Ovid MEDLINE(R) Search Strategy December 8, 2022

Research question: *What has been reported in the literature on the intersections of poverty and grief due to the death of a family member or friend in college students of any age who identify or are identified as non-white racial or ethnic groups?*
Secondary research question: *What interventional supports are in practice on or off campus, and what supports are recommended for these students to support their academic performance and degree completion?*

Tables of search concepts and search statements:

**Concept**	**Statement #**
Grief	#1
Death + family or friends	#6
Students	#12 or #13
Poverty	#16
Race or minorities	#25
**Combination sets, combined with OR**	
Grief + Students Set	1 and (or/12-13)
Death + Students + Poverty or Minorities	(#6 and 12) and (#16 or #25)
**Note:** + indicates the AND connector	

Ovid MEDLINE(R) and Epub Ahead of Print, In-Process, In-Data-Review & Other Non-Indexed Citations, Daily and Versions <1946 to December 08, 2022>

1 bereavement/ or grief/ or disenfranchised grief/ or prolonged grief disorder/ or (bereav* or grief* or griev* or mourn*).ti,ab,kf,kw. [Grief subject/keyword terms] 23764
2 Death/ or (death or near-death or orphan* or thanato*).ti,ab,kf,kw. [Death subject/keyword terms] 873216
3 exp family/ or exp family characteristics/ or friends/ [Family/friends subject terms] 366205
4 and/2-3 [Death + Family/Friends subject terms] 16477
5 ((loss or death or deceased) adj6 (family* or families or familial or household or stepfamil* or step-famil* or kinship* or filiation or relatives or "blood relative*or parent" or parents or parental or "single parent" or single-parent* or "one parent*" or one-parent* or grandparent* or grandmother* or grandfather* or great-grandparent* or great-grandmother* or great-grandfather* or child* or maternal or mother* or paternal or father* or stepparent* or stepfather* or stepmother* or stepchild* or step-parent* or step-father* or step-mother* or step-child* or spouse* or spousal or husband* or wife or wives or "domestic partner*" or sibling* or sister* or brother* or son or sons or daughter* or grandson* or granddaughter* or grand-child* or grandchild* or aunt* or uncle* or niece* or nephew* or cousin* or friend or friends or acquaintance* or companion* or classmate or "class mate*" or roommate* or "room mate*" or boyfriend* or "boy friend*" or girlfriend* or "girl friend*")).ti,ab,kf,kw. [Death family/friend keywords] 53826
6 or/4-5 [Death - family/friends set] 64458

7 students, health occupations/ or students, dental/ or students, medical/ or students, nursing/ or students, pharmacy/ or students, premedical/ or students, public health/ [health students - subject terms] 84581
8 (students/ or student dropouts/) and (library schools/ or schools, health occupations/ or area health education centers/ or schools, dental/ or schools, medical/ or schools, nursing/ or schools, pharmacy/ or schools, public health/ or schools, veterinary/ or universities/) [students + colleges subject terms] 22607
9 (((student* or students* or apprentice* or trainee*) and (college* or colleges or university* or universities or doctoral or graduate or nontraditional or non-traditional or postgrad* or post-grad* or undergrad*)) or "adult learner*").ti,ab,kf,kw. [college student keywords] 137900
10 ((college* or colleges or university* or universities) adj3 (freshman or freshmen or junior or juniors or senior or seniors or sophomore or sophomores)).ti,ab,kf,kw. [college student keywords 3] 1944
11 ((student* or students* or apprentice* or trainee*) adj3 (dental or medical or nursing or pharmacy or premedical or "public health" or veterinary)).ti,ab,kf,kw. [college student keywords 2] 92245
12 or/7-11 [Students set 1 - college students] 226026
13 students/ or student dropouts/ [Student subject terms 2] 78244
14 poverty/ or child poverty/ or sociodemographic factors/ or socioeconomic factors/ or economic factors/ or economic stability/ or economics/ or economics.fs. or housing instability/ or economic status/ [Poverty subject terms] 643208
15 (poverty* or indigen* or "low* income" or low-income or lower-income or socioeconomic* or ((food or housing or economic* or financial or income or salar* or wage* or pay) adj2 (insecur* or secur* or instability* or stability* or inequalit* or inequit* or disparit*))).ti,ab,kf,kw. [poverty keyword terms] 258666
16 or/14-15 [Poverty set] 816031
17 race factors/ or racism/ or race relations/ or apartheid/ or desegregation/ [Race Subject terms] 9257
18 (racism* or racist* or racial* or race-related or interracial* or "inter racial*" or multiracial* or "multi racial" or biracial or "bi racial*" or anti-racis* or anti-racial* or antiracis* or antiracial* or ((race or races) adj6 (discriminat* or factor or factors or group* or relation*)) or apartheid* or desegregat* or de-segregat* or segregat*).ti,ab,hw,kf,kw. [Race/racism keywords] 183637
19 continental population groups/ or african continental ancestry group/ or african americans/ or american native continental ancestry group/ or indians, central american/ or indians, north american/ or alaskan natives/ or indigenous canadians/ or inuits/ or american natives/ or indians, south american/ or asian continental ancestry group/ or asian americans/ or european continental ancestry group/ or oceanic ancestry group/ [Subject terms for racial groups] 238490
20 (((African or Arab or Asian or Black or Chinese or Cuban or Indian* or Japanese or Korean or Mexican or native or "Puerto Rican*" or Spanish) adj2 American*) or ((African or Arab or Asian or Oceanic or Pacific) adj2 ancestry) or Blacks or Chicana* or Chicano* or Hispanic* or Latino* or Latina* or Latinx or ((Alaska* or American or Hawaiian*) adj2 native*) or indignenous* or "first nation" or "native people*" or "native person*" or native-born or tribe or tribesor ethno-rac* or ethnorac* or nonwhite* or non-white*).ti,ab,hw,kf,kw. [Ethno-racial groups - keywords] 252460

21 ethnic groups/ or african americans/ or amish/ or arabs/ or asian americans/ or indigenous peoples/ or alaskan natives/ or inuits/ or roma/ or "Hispanic or Latino"/ or mexican americans/ or jews/ or (amish or inuit or inuits or roma or jew or jewish or jews or ethnic* or ethnicity-based or ethnolog* or nationalit*).ti,ab,hw,kf,kw. [ethic groups subject/keywords] 312723

22 Healthcare disparities/ or health status disparities/ or health services accessibility/ or health equity/ or right to health/ or ("right to health*" or (health* adj2 (access* or disparit* or equit* or inequal* or inequit*))).ti,ab,kf,kw. [Disparities subject/keyword terms] 171285

23 minority health/ or minority groups/ or minorit*.ti. or (minorit* adj2 (group* or health)).ti,ab,kf,kw. [minority subject/keyword terms] 30351

24 Vulnerable Populations/ or (vulnerable* adj2 (people* or person* or population*)).ti,ab,kf,kw. [vulnerable subject/keyword terms] 30839

25 or/17-24 [Minorities set] 777224

26 ("32664785" or "27132381" or "28198653" or "15802260" or "18680889" or "33669340" or "35435135" or "35992443" or "34162233" or "33505337" or "30376585").ui. [11 exemplars with students] 11

27 1 and (or/12-13) [Grief + Students Set] 548

28 (and/6,12) and (or/16,25) [Death + Students 1 + Poverty or Minorities] 53

29 or/26-27 [Grief+Students final set finds 11 exemplars] 548

30 or/27-28 [Final set] 597

Medline (Ovid) legend:
Field codes: / = Medical Subject Heading (MeSH); ti = title; ab = abstract; hw = subject heading word; kf = keyword heading word; kw = keyword heading (author keywords); ui = unique identifier
Proximity operator: adj#
Truncation: *
