# Supplementary material for: Exploring the intersections of college student poverty, grief, and racial/ethnic identity: a scoping review protocol
Source: Syst Rev. 2023 Dec 14;12:235. doi: 10.1186/s13643-023-02407-x (PMC10720161; doi:10.1186/s13643-023-02407-x)
Supplement: Supplementary file 3 — Additional file 3. Draft Data Chart. [file 13643_2023_2407_MOESM3_ESM.pdf]

Exploring the intersections of college student poverty, grief, and racial/ethnic identity: a scoping review protocol

Additional file 3. Draft Data Chart

**Reviewer:**	
**Date:**	
**Key elements:**	**Reviewer's response:**
Author(s), year, country where research was conducted.	
Research design.	
Aim/purpose.	
**Participants** (College students of any age with racial or ethnic identities other than White).	
Study population, sample size, and time frame of study.	
Racial or ethnic identity, self or other defined.	
Demographic profile (race, ethnic identity, age, gender, student status, socio-economic status, etc.).	
Year of college. Type of college.	
Data collection/recruitment procedure.	
**Concept** (Experiences of poverty and grief due to the death of a family member or friend, as well as interventional support for academic performance and degree completion).	
Includes definition of poverty and/or food, financial, and housing insecurity (Y/N).	
Includes definition of grief (Y/N).	
Measures/screening tools used within the model of care for poverty, grief due to the death of a family member or friend, and demographic information.	
Intervention method (case management, referrals, consultations, group participation, psychotherapy, etc.).	
Outcomes (academic performance and degree completion).	
**Context** (Any geographic setting worldwide. Interventional support can be delivered from campus or community services, on or off-campus locations).	
Location of services (onsite college/university or community care providers).	
